# Study on the Filtration Performance of the Baghouse Filters for Ultra-Low Emission as a Function of Filter Pore Size and Fiber Diameter

**DOI:** 10.3390/ijerph16020247

**Published:** 2019-01-16

**Authors:** Xingcheng Liu, Henggen Shen, Xueli Nie

**Affiliations:** School of Environmental Science and Engineering, Donghua University, Shanghai 201620, China; shenhg@dhu.edu.cn (H.S.); 2121187@mail.dhu.edu.cn (X.N.)

**Keywords:** PM_2.5_, air pollution, ultra-low emission, baghouse filter, filter pore size, fiber diameter, filtration performance

## Abstract

The main objective of this study was to determine the effect of filter pore size and fiber diameter on the performance of the baghouse filters for ultra-low emission. In this study, three kinds of conventional polyester filter (depth filtration media) and two kinds of polytetrafluoroethylene membrane-coated polyester filter (surface filtration media), having various filter pore sizes and fiber diameters, were tested to determine the performance of static and dynamic filtration. In order to determine the static filtration performance, the filtration resistance and the filtration efficiency of the clean filter media were measured by the arrestance method. The dynamic filtration performance experiments were conducted to determine the dynamic resistances, dust depositions, and dynamic filtration efficiencies of the dust-containing filter media under the condition of dust airflow filtration through a pulse-cleaning cycle. In the dynamic filtration performance experiments, the size of 50% test dust was less than 2.5 μm, and the mass mean aerodynamic diameter of the dust was 1.5 μm. The filtration velocity was 2 m∙min^−1^, and the dust concentration was 18.4 g∙m^−3^. The static filtration performance experiments showed that the filter pore size greatly affected the filtration resistance and the filtration efficiency of the fabric structure of the surface filtration media. In the depth filtration media, the filtration efficiency and the filtration resistance of the fabric structure were improved when the filter pore size and the fiber diameter were smaller in magnitude. For all the five filter media, smaller the pore size of the filter media, greater was the filtration precision (for fine particles, such as PM_2.5_) of the fabric structure. In the dynamic filtration performance experiments, the filter pore size and the fiber diameter of the depth filtration media affected the dynamic filtration resistance and the dynamic filtration efficiency of the depth filtration media by affecting the deposition rate of dust in the depth filtration media; however, the filter pore size of the surface filtration media affected the blocking rate of dust in the membrane micropores, thus influencing the dynamic filtration resistance and the dynamic filtration efficiency of the surface filtration media.

## 1. Introduction

Baghouses are widely used to purify the flue gases emitted by industries. They represent some of the most important equipment used for environmental protection. They are used for the efficient removal of industrial dusts, especially fine particles [1,2,3]. The operation of a baghouse filter can be described as follows: when the airflow containing dust passes through the filter, the particles are captured and a dust cake is formed on the surface of the filter [4,5,6,7]. The formation of a dust cake on a conventional filter media has to go through three stages: depth filtration, transient filtration, and surface filtration [8,9]. The membrane-coated filter has a layer of microporous polytetrafluoroethylene (PTFE) membrane on its surface, enabling the direct passage of dust into the surface filtration stage [10,11]. Therefore, the deposition morphology of dust is different in a needle-punched/spunlaced filter (conventional filter) and in a membrane-coated filter. Conventional filters are generally known as depth filtration media, while membrane-coated filters are generally known as surface filtration media in the industry.

A filter medium is the core component of a baghouse; its filtration performance will directly affect the operation energy consumption and the dust collection efficiency of a baghouse. Several research studies have been conducted to determine the filtration performance of filter media. In these studies, filtration resistance, filtration efficiency, and cleaning performance were determined [12,13,14,15,16]. Sang et al. [17] determined whether the processing conditions were affected by the filtration performance of filter bags. The results indicated that the differential pressure of the filter bags decreased while the collection efficiency increased with an increase in main-needling strokes. Jiang et al. [18] determined the filtration characteristics of a PTFE membrane-coated filter medium. Compared to a needle-punched filter medium, the filtration efficiency of a PTFE membrane-coated filter medium was greater than 99.99% for micron-sized particles. Liu et al. [19] investigated the relationship between the filtration efficiency, filtration velocity, and particle sizes of a PTFE membrane-coated filter medium. The efficiency curves for PTFE membrane-coated filter media are typically “v” shaped for the following characteristics: the particle sizes are in the range of 10 to 300 nm and face velocities are in the range of 0.3 to 15 cm∙s^−1^. For PTFE membrane-coated filter media, the most penetrating particle sizes (MPPS) decrease gradually with an increase in the face velocity. Bao et al. [20] determined the influence of fibers on the dust dislodgement efficiency of filter bags. The authors concluded that the dust could be dislodged more easily from the filter when the linear density of the fiber in the filter bag was higher. When the Young’s modulus had a lower value, the fiber could be bent easily and the dust could be prevented from penetrating the filter. Cirqueira et al. [21] performed surface treatment of a conventional needle-punched filter to avoid the deep deposition of particles. With this strategy, the filter cleaning cycle was prolonged and the filtration efficiency of the filter media was improved. However, previous scholars did not study the differences in the deposition morphology of dust, which was associated with the surfaces of the depth filtration media and the surface filtration media. On the other hand, there are few reports about the effects of filter pore size and fiber diameter on the deposition morphology of dust in the two types of filter media and the filtration performance of the two types of filter media.

In this experimental study, we investigated the influence of filter pore size and fiber diameter on the filtration resistance and filtration efficiency of clean filter media. We also investigated the effect of filter pore size and fiber diameter of a baghouse filter on the dust deposition rate, dynamic filtration resistance, and dynamic filtration efficiency. For this purpose, static and dynamic filtration performance tests were conducted on three kinds of conventional polyester filter media (depth filtration media) and two kinds of PTFE membrane-coated polyester filter media (surface filtration media).

## 2. Materials and Methods

### 2.1. Filter Samples

Table 1 displays performance parameters of the five kinds of polyester filters which were included in this study. All five filter media were manufactured by Aobo Technology Co., Ltd. (Dezhou, Shandong, China). The three kinds of depth filtration media were as follows: polyester spunlaced felt (PSF), polyester needle-punched felt (PNF), and Aobo polyester needle-punched felt (APNF). The two kinds of surface filtration media were Aobo polyester membrane-coated needle-punched felt (APMCNF) and polyester membrane-coated needle-punched felt (PMCNF). The two kinds of Aobo filter media (APNF, APMCNF) were composed of polyester fibers of different fiber diameters (95 wt% 16-μm-diameter fiber and 5 wt% 28-μm-diameter fiber). The remaining three kinds of filter media were composed of polyester fiber of the same fiber diameter (fiber diameter = 16 μm). Using a PTFE membrane-coated process, we developed the surface filtration media of APMCNF and PMCNF from the depth filtration media of APNF and PNF, respectively.

**Figure 1:**
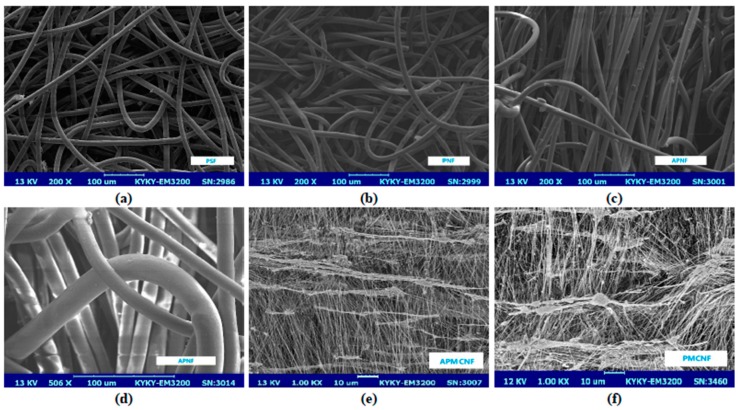
SEM micrographs of the filter surfaces: (**a**) PSF, (**b**) PNF, (**c**) APNF, (**d**) APNF (Fibers of different diameters), (**e**) APMCNF, (**f**) PMCNF.

Figure 1 illustrates SEM images of the surfaces of the five kinds of filter media. Figure 1a–c are the three kinds of depth filtration media. Figure 1d shows the fibers of different diameters in APNF filter media. Figure 1e,f are the two kinds of surface filtration media. From Figure 1, it can be seen that unlike the depth filtration media, the surface filtration media is coated with a microporous membrane, which makes it difficult for the dust to enter the interior of the filter media, and the filtration effect of the filter media on the dust mostly occurs on the microporous membrane of the surface filtration media.

For the three kinds of depth filtration media, the average pore size of the filter media is closely related to the filtration efficiency. The fiber diameter of the filter media directly affects the deposition mechanism (interception), and surface growth of the deposited area [22]. Therefore, PSF and PNF filter media were used to investigate the influence of the filter pore size on the filtration performance of the depth filtration media. APNF filter media and PNF filter media were used to study the effect of the fiber diameter on the filtration performance of the depth filtration media.

For the two kinds of surface filtration media, the membrane of the filter media is the main structure to perform the filtration function, the fiber layer mainly plays the role of supporting the membrane (as shown in Figure 1e,f). The pore size of the filter media is the main factor affecting the filtration performance. Therefore, APMCNF filter media and PMCNF filter media were used to investigate the effect of filter pore size on the filtration performance of the surface filtration media.

The fiber-filling rate and the filter pore size of the five kinds of filter media were measured with a capillary flow porometer (PMI capillary flow porometer, Porous Materials, Inc., Ithaca, NY, USA). Since the pore size and distribution of the filter media were measured by the bubble-point test method, the fiber-filling rate of the surface filtration media measured by the capillary flow porometer cannot reflect the true situation of the surface filtration media. Therefore, the fiber-filling rates of the two surface filtration media are not listed in Table 1, but this does not hinder the study of the effect of filter pore size on the filtration performance of the surface filtration media. The diameter of fibers was measured with a scanning electron microscope (SEM) (Model KYKY-EM3200, KYKY Technology Co., Ltd., Beijing, China) and a fiber fineness analyzer (Model XGD-1, Xinxian Co., Ltd., Shanghai, China).

### 2.2. Static Filtration Performance Experiment

**Figure 2:**
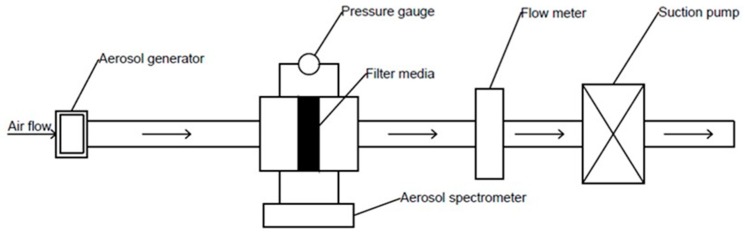
An experimental device was used to determine the static filtration performance of a filter media.

In the experiment used to determine the static filtration performance, the filtration resistance and the filtration efficiency of the clean filter were measured by the arrestance method. The experimental results indicate the influence of the filter pore size and the fiber diameter on the filtration resistance and the filtration efficiency of the fabric structure of a filter media. Figure 2 illustrates the performance of the static filtration device, which was used in this experiment: an aerosol generator (Model 8108, TSI, Inc., Shoreview, MN, USA) was used to produce aerosol particles from potassium chloride. The size of aerosol particles was in the range of 0.1–10 μm. The filter media was supported by a filter holder. Two aerosol spectrometers (Model 1.108, GRIMM Aerosol Technik Ainring GmbH & CO.KG, Ainring, Bayern, Germany) were used to determine the mass concentration of aerosol particles, which moved upstream and downstream of the filter media. A pressure gauge was used to determine the filter’s pressure drop (The pressure drop of the filter media represents the filtration resistance of the filter media.); the face velocity was controlled by a flow meter. The temperature of the laboratory was maintained at 20 °C, while the relative humidity was kept fixed at 60%. Equation (1) was used to calculate the static filtration efficiency ($\eta_{s}$) of the filter media:

(1) $$\eta_{s}=\frac{C_{i}-C_{f}}{C_{i}}\times 100\%$$

Here, $C_{i}$ and $C_{f}$ are the particle mass concentrations in the front and rear airflow of the filter media, respectively.

### 2.3. Dynamic Filtration Performance Experiment

**Figure 3:**
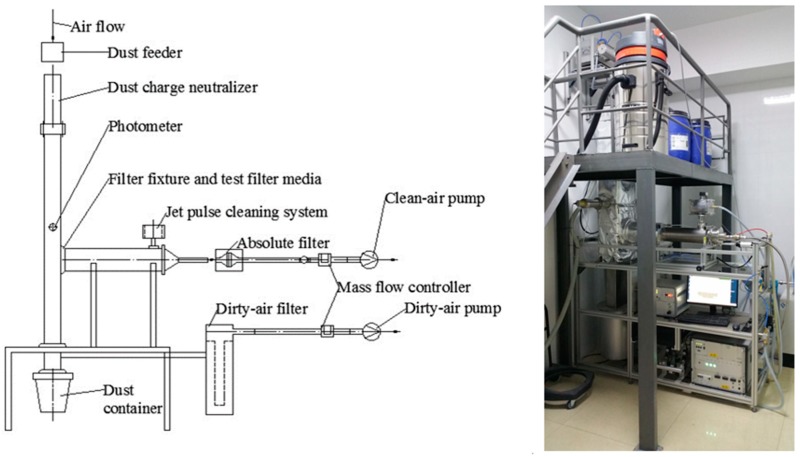
An experimental device was used to determine the dynamic filtration performance of a filter media.

In a laboratory, an experiment was conducted to determine the dynamic filtration performance of a baghouse filter. In this experiment, we simulated the working conditions of particulate matter filtration and filter media regeneration in a dust-laden airflow. The filtration performance parameters, such as dynamic filtration resistance, dynamic filtration efficiency, and dust deposition of the filter media, were assessed. The results of dynamic filtration experiments reflected the influence of filter pore size and fiber diameter of the dust-containing filter media on the deposition rate of particulate matter, the formation of the dust cake, the dynamic filtration resistance, and the dynamic filtration efficiency. Based on the ASTM D6830-02 standard, we performed dynamic filtration experiments on the filter media by using a gas filtration device and a pulse reverse blowing system. Figure 3 illustrates that the experimental device includes the following components: a dust feeder, a dust charge neutralizer, a photometer, a filter media fixture, a jet pulse cleaning system, an absolute filter, and a data acquisition system to indicate pressure drop.

An experimental device was developed to measure the dynamic filtration performance of a baghouse filter. This experimental device was based on the following principle: in the dust feeder, the dust was dispersed into the compressed air to form a uniform concentration of dust (the dust concentration was fixed at 18.4 g∙m^–3^). Then, the dust was evenly distributed throughout the vertical pipe and the down flow. Some parts of the dust-laden airflow reached the horizontal pipe, and it then passed through the tested filter media that was operated at a filtration velocity of 2 m∙min^–1^. The diameter of the filter media was 150 mm. To simulate the operating conditions, we exposed the filter media to a constant concentration of dust-laden airflow. Meanwhile, an absolute filter was used to collect the particulates that penetrated the filter media. A dust cake was formed by the filtering dust, leading to an increase in the pressure drop of the filter media. The differential pressure sensor was used to detect and record the pressure drop both before and after the filter media. No sooner did the pressure drop reach the preset value of 1000 Pa, the system released pulses of a compressed air stream (cleaning pressure = 0.5 MPa, pulse time = 50 ms) to clean the filter media. After the completion of the pulse action, the next filtration cycle was started and continued until the differential pressure reached 1000 Pa. The next pulse was thus initiated with this process. This is also known as the normal filtration cycle. In the experiments used to determine dynamic filtration performance, the test dust consisted of Pural NF alumina particle. In the test dust, the size of 50% particles was less than 2.5 μm, and the mass mean aerodynamic diameter of particles was 1.5 μm (D_50_ = 1.5 μm). The temperature of the laboratory was maintained at 20 °C, and the relative humidity was fixed at 60%. According to the test requirements of ASTM D6830-02 standard, we divided the experiment of dynamic filtration performance into three phases:

(A) Fabric conditioning period (aging phase): The period during which the filter media was conditioned within a test apparatus by subjecting it to 10,000 cleaning pulses of rapidly compressed air. These pulses were spaced out from each other by five seconds. The residual resistance drop (the filtration resistance was measured across the test filter medium three seconds after cleaning the test filter media with a pulse of compressed air; it is referred to as residual differential pressure) $\Delta P_{r1}$ was recorded after each cleaning. After the completion of the aging operation of the filter media, both the test filter media and the absolute filter media were removed and weighed. The gain in filter media mass in the aging phase was calculated according to Equation (2). During the fabric conditioning period, the dynamic filtration efficiency ($\eta_{1}$) of the filter media was calculated according to Equation (3):

(2) $$\Delta m=M_{2}-M_{1}$$

Here, *M*_1_ and *M*_2_ represent the mass of the filter media before and after the aging phase, respectively.

(3) $$\eta_{1}=\left(1-\frac{m_{12}-m_{11}}{50000\times c\times Q}\right)\times 100\%$$

Here, *m*_11_ and *m*_12_ represented the mass of the absolute filter before and after the aging phase, respectively. *C* is the concentration of the dust, and *Q* is the flow rate of clean-air pump.

(B) Fabric recovery period (recovery phase): The dust filtering operation was continued for the aged filter media. When the filter’s pressure reached 1000 Pa, the filter medium was cleaned by compressed air cleaning pulses. A normal filtration cycle was repeated 30 times. The residual resistance drop ($\Delta P_{r2}$) of the filter media was recorded after each cleaning cycle. After subtracting the weight of the absolute filter, the dynamic filtration efficiency (*η*_2_) of the filter media was calculated using Equation (4):

(4) $$\eta_{2}=\left(1-\frac{m_{22}-m_{21}}{t_{2}\times c\times Q}\right)\times 100\%$$

Here, *m*_21_ and *m*_22_ represented the mass of the absolute filter both before and after the recovery phase, respectively.

(C) Performance test period (measurement phase): Remove the absolute filter, and replace it with a PM_2.5_ separator. In accordance with step B, the dust filtering operation was carried out for six hours through continuous normal filtration cycle. The residual resistance drop ($\Delta P_{r3}$) of the filter media was recorded after each cleaning cycle. The dynamic filtration efficiencies of PM_2.5_ ($\eta_{3}$) and TOT (particle size > 2.5 μm) ($\eta_{4}$) were calculated by using Equations (5) and (6), respectively:

(5) $$\eta_{3}=\left(1-\frac{m_{32}-m_{31}}{21600\times c\times Q}\right)\times 100\%$$

(6) $$\eta_{4}=\left(1-\frac{m_{32}^{\prime }-m_{31}^{\prime }}{21600\times c\times Q}\right)\times 100\%$$

Here, *m*_31_ and *m*_32_ denoted the mass of PM_2.5_ absolute filter before and after the measurement phase, respectively. Furthermore, $m_{31}^{\prime }$ and $m_{32}^{\prime }$ denoted the mass of TOT absolute filter before and after the measurement phase, respectively.

## 3. Results and Discussion

### 3.1. Static Filtration Performance

#### 3.1.1. Filtration Resistance

**Figure 4:**
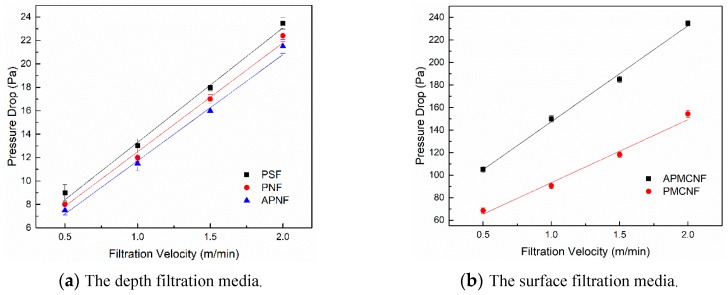
The pressure drop was plotted as a function of the filtration velocity of the clean filter media.

Figure 4 shows the changes in the filtration resistance with respect to the filtration velocity of the five kinds of filter media, which were maintained in a clean state. The filtration resistances of the five filter media increased with an increase in filtration velocity. The filtration resistances of the two surface filtration media were much greater than that of the remaining three depth filtration media, which was consistent with the results of literature [23,24]. When the filtration velocity was kept fixed at 0.5 m∙min^–1^, the filtration resistances of the five filter media (PSF, PNF, APNF, APMCNF, and PMCNF) were 9.0, 8.1, 7.3, 105.5, and 68.5 Pa, respectively. When the filtration velocity was kept fixed at 2 m∙min^–1^, the filtration resistances of the five filter media were 23.5, 22.4, 21.7, 235.2, and 154.4 Pa, respectively. With an increase in the filtration velocity, we observed a slight change in the gaps of filtration resistances that occurred between the three depth filtration media. With an increase in filtration velocity, there was an increase in the gaps of filtration resistances between the two surface filtration media.

Figure 4a illustrates that under the clean state, the filtration resistance of the fabric structure of PSF filter media was greater than that of PNF filter media. Moreover, the fiber diameter (16 μm), the thickness of the filter, and the fiber-filling rate of PSF filter media were the same as that of PNF filter media. However, the average pore size of PSF filter media (18.26 μm) was less than that of PNF filter media (22.49 μm). This indicates that when the filter pore size was smaller, we could increase the filtration resistance of the fabric structure of the depth filtration media. The filter pore size of PNF filter media was similar to that of APNF filter media (22.52 μm); however, the fiber diameter of APNF filter media were different from that of PNF filter media. Nevertheless, the filtration resistance of PNF filter media was greater than that of APNF filter media. The experimental results indicate that larger the fiber diameter of the depth filtration media, lower would be the filtration resistance of the fabric structure of the depth filtration media.

Figure 4b shows that the filtration resistance of the two surface filtration media exhibited the following trend: APMCNF > PMCNF. The average pore sizes of the two filter media were as follows: APMCNF (0.42 μm) < PMCNF (1.05 μm). Unlike the depth filtration media, the surface filtration media mainly depend on the membrane to filter dust. The filtration resistance of the fabric structure of the surface filtration media was mainly affected by the size and distribution of the filter pores; moreover, the fiber of the fabric played an important role in supporting the membrane. Therefore, the fiber diameter and fiber-filling rate are not the main factors that affect the filtration resistance and filtration efficiency of the fabric structure of the surface filtration media. Consequently, it can be concluded that when the average pore size of the surface filtration media is smaller, the filtration resistance of the fabric structure of the surface filtration media is greater.

#### 3.1.2. Filtration Efficiency

When polydisperse particles were filtered by a fiber filter media, smaller particles got deposited on the fiber surface through diffusion. Meanwhile, larger particles were mainly deposited through interception and inertia. With an increase in particle size, the diffusion efficiency of the filter media weakened gradually with respect to the particulate matter. Meanwhile, the interception and inertial efficiencies increased gradually. The filter media would have a minimum filtration efficiency when the particle size was varied between the upper limit of filter media diffusion and the lower limit of interception. The particle size that corresponded with this minimum value is known as the most penetrating particle size (MPPS) [25].

**Figure 5:**
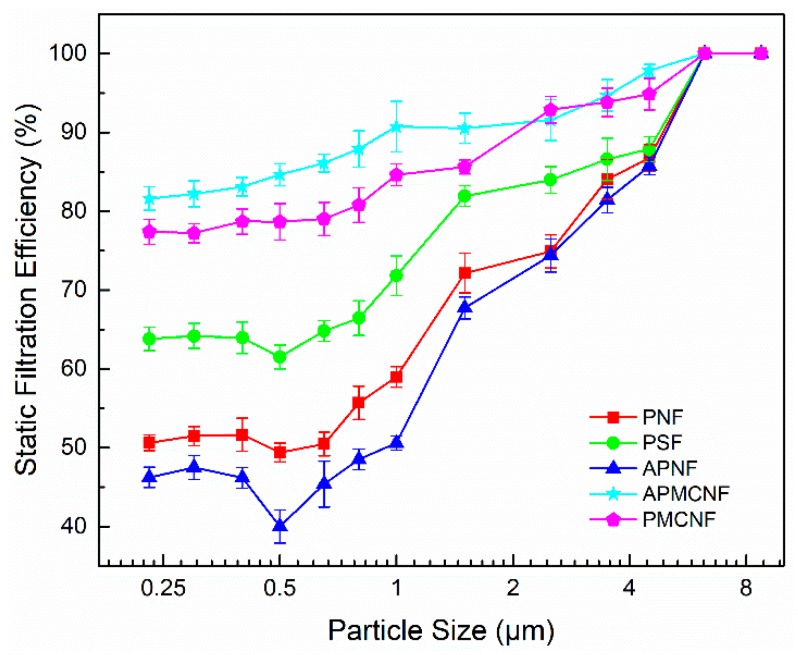
The classification filtration efficiencies of the clean filter media for particles at a filtration velocity of 1 m·min^−1^.

Figure 5 illustrates the classification filtration efficiencies of the clean filter media for different particle sizes when the filtration velocity was kept intact at 1 m∙min^–1^. The classification filtration efficiencies of the five kinds of filter media increased with an increase in particle sizes. For particles whose size ≥ 6 μm, the filtration efficiencies of the five filter media were 100%. When the particle size < 6 μm, the filtration efficiencies of the two surface filtration media were greater than the remaining three depth filtration media. When the average pore size of the five clean filter media was decreased, there was an increase in the filtration efficiencies of the filter media. For the depth filtration media, the fluctuation in the filtration efficiencies was greater than that of the surface filtration media. In particular, this trend was true for APNF filter media. When the filtration efficiency curve of APNF filter media was constructed, an obvious “V” shaped trough was formed. In addition, the three depth filtration media showed the lowest filtration efficiency when the particle size was about 0.5 μm. Under the conditions of this experiment, the MPPS of the three depth filtration media was about 0.5 μm. In this experiment, we did not measure the MPPS of the two surface filtration media. Previous literature [19,26] studies indicate that MPPS also existed in a membrane-coated filter media. The value of MPPS was in the range of 0.03–0.1 μm, which exceeded the measurable precision range (0.23–30 μm) of the aerosol spectrometer and the range of particle size (0.1–10 μm) that was produced by the aerosol generator. The filtration efficiency experiments showed that the MPPS of the surface filtration media was smaller than that of the depth filtration media. Moreover, the MPPS depended on the difference between the average pore sizes of the two types of filter media.

**Figure 6:**
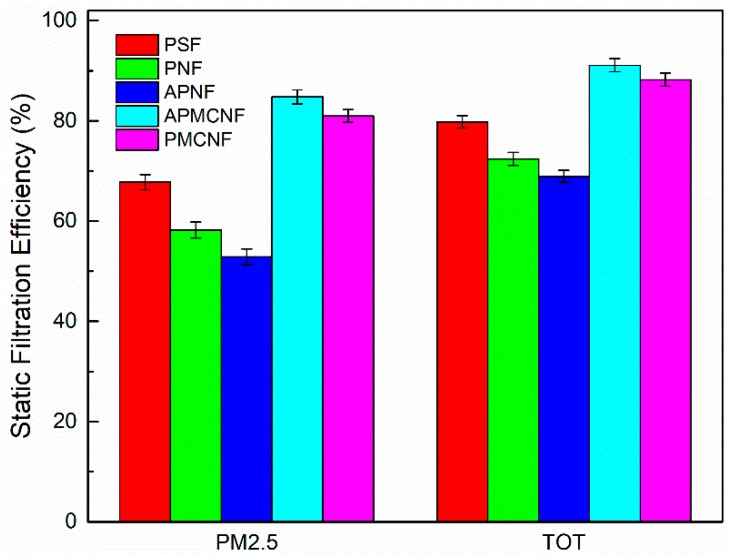
The static filtration efficiencies of the clean filter media for PM_2.5_ and TOT at a filtration velocity of 1 m·min^−1^

Figure 6 shows the static filtration efficiencies of the five clean filter media on PM_2.5_ and TOT; the filtration velocity was maintained at 1 m∙min^–1^. The filtration efficiencies of the two surface filtration media were greater than 80% for PM_2.5_. For the depth filtration media of PM_2.5_, the filtration efficiencies were 67.9% (PSF), 57.4% (PNF), and 51.3% (APNF). The filtration efficiencies of the two surface filtration media were greater than 87% for TOT. The filtration efficiencies of the remaining three filter media were 79.8% (PSF), 72.4% (PNF), and 68.8% (APNF). By performing the static filtration experiments on fine particles, we found that smaller the average pore size of the filter media, greater was the filtration precision of the clean filter media. In addition, the filter pore size and the fiber-filling rate of APNF and PNF filter media were similar; however, the filtration efficiency of PNF filter was obviously greater than that of APNF filter media. This indicates that the filtration efficiency of the fabric structure of the depth filtration media could be improved by decreasing the fiber diameter.

### 3.2. Dynamic Filtration Performance

#### 3.2.1. Dynamic Filtration Resistance

In the dynamic filtration experiment, the residual resistance drop was considered to be an important index of the filtration performance. The variation in the residual resistance drop depends on the amount of dust deposition on the surface or inside the filter media. Based on the test requirements of ASTM-D6830 standard, the dynamic filtration test was divided into three phases: aging phase, recovery phase, and measurement phase. Regardless of a variety of testing times and the filtration cycles of a filter media, consistency was only observed in the aging phase (cleaning was conducted once every 5 s for a total of 10,000 filtration cycles). Therefore, the increase rate of the residual resistance drop in the aging phase can reflect the rate of dust deposition on the surface or inside the different filter media. During the aging phase, there were 10,000 filtration cycles of each filter media. To facilitate lawful analysis, we divided 10,000 filtration cycles into 20 intervals of the aging phase that were associated with each filter media. Each interval contained the data of 500 residual resistance drops. The average value of these 500 residual resistance drops represented the residual resistance drop of the interval.

**Figure 7:**
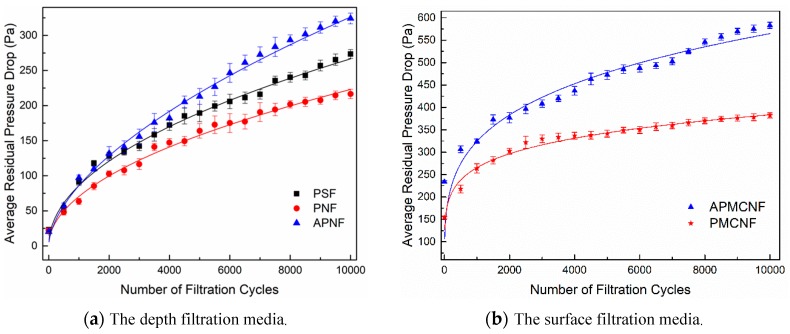
The changes in the average residual resistance drop of the five filter media during the fabric conditioning period.

Figure 7a illustrates that the average residual resistance drops of the three filter media increased exponentially when the filtration cycle was increased for the depth filtration media (PSF, PNF, and APNF). This indicates that in the dynamic filtration process, the filter media could be cleaned and regenerated; however, a foundation structure of the stable dust cake could still be formed inside the depth filtration media. With a continuous process of filtration, the structure of the dust cake could be increased continuously inside the filter media. This led to a continuous increase in the residual resistance drop. In the three depth filtration media, the average residual resistance drops increased in the following order: APNF > PSF > PNF. The initial filtration resistance drop of the three filter media were equivalent. At the end of the first 500 filtration cycles, the average residual resistance drops of the three filter media were as follows: 54.7 Pa (PSF), 48.2 Pa (PNF), and 57.9 Pa (APNF). At the end of the aging phase, the final average residual resistance drops of three filter media were as follows: 273.6 Pa (PSF), 216.7Pa (PNF), and 324.2 Pa (APNF). The difference between average residual resistance drops of the three depth filtration media reflected the different dust deposition conditions in the three depth filtration media. In this experiment, both the PSF filter media and PNF filter media had the same fiber diameter, but the average pore size of PNF filter media was greater than that of PSF filter media. For PNF filter media, the increase in the rate of average residual resistance drop during the aging phase was less than that of PSF filter media. This indicates that when the fiber diameter was constant, smaller the average pore size of the depth filtration media, the faster would be the dust deposition rate of depth filtration media, and greater would be the amount of dust deposited in the depth filtration media. The average pore size of PNF filter media was 22.49 μm, while the average pore size of APNF filter media was 22.52 μm. This indicates that the average pore size of APNF filter media was similar to that of PNF filter media; however, the increasing rate of the average residual resistance drop of APNF filter media was the highest among the three depth filtration media. This may be attributed to the fact that the fiber diameter of APNF filter media was larger. Larger the fiber diameter, greater would be the chances and the probability of dust deposition on the surface of the fiber. These conditions were more favorable for the deposition of dust in the depth filtration media, and it led to the formation of a dust cake.

**Figure 8:**
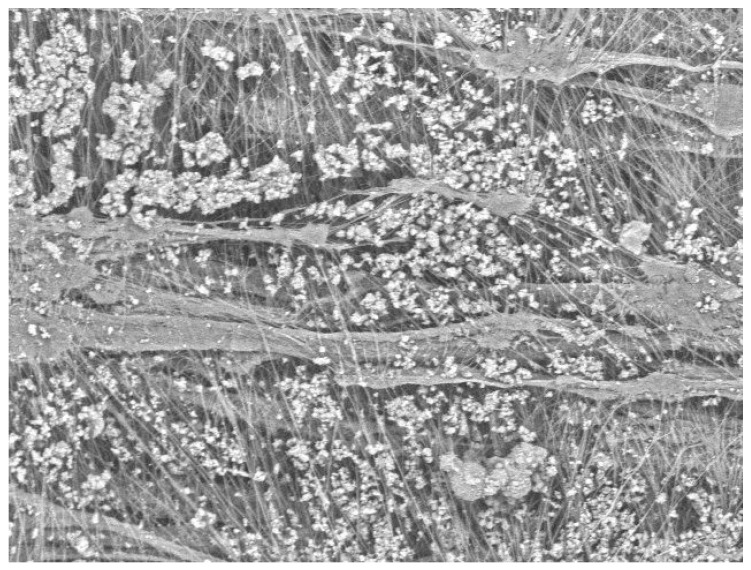
The adhesion of dust on the surface of APMCNF filter media.

Unlike the depth filtration media, it was difficult for the dust to pass into the surface filtration media and form a dust cake. Most of the dust particles could only deposit on the surface of the membrane. For the surface filtration media, the fiber diameter was not the main factor that affected the deposition of dust. Moreover, the surface of the membrane-coated filter media was smooth and could be cleaned easily; therefore, it was difficult to form a stable layer of dust on the surface of the membrane-coated filter media during the process of dynamic filtration. The dynamic filtration resistance of the surface filtration media was mainly governed by the size and distribution of membrane pores. Figure 7b illustrates that the average residual resistance drops of the two surface filtration media (APMCNF and PMCNF) increased exponentially with an increase in filtration cycles. For APMCNF filter media, the increasing rate of average residual resistance drop was greater than that of PMCNF filter media. At the end of the first 500 filtration cycles, the average residual resistance drops of the two filter media were as follows: 306.7 Pa (APMCNF) and 218.1 Pa (PMCNF). At the end of the aging phase, the final average residual resistance drops of the two surface filtration media were 583.5 Pa (APMCNF) and 382.3 Pa (PMCNF). The difference between the average residual resistance drops of the two filter media increased with the filtration process. The average pore sizes of the two filter media were as follows: 0.42 μm (APMCNF) and 1.05 μm (PMCNF). This indicates that smaller the average pore size of the surface filtration media, greater would be the dynamic filtration resistance. Figure 8 illustrates that the average residual resistance drop of the surface filtration media increased when the dust blocked the pores of the filter membrane. The experimental results indicate that dust can easily block the micropores of the surface membrane when the average pore size of the surface filtration media is smaller. Consequently, the average residual resistance drop of the surface filtration media increased rapidly.

**Figure 9:**
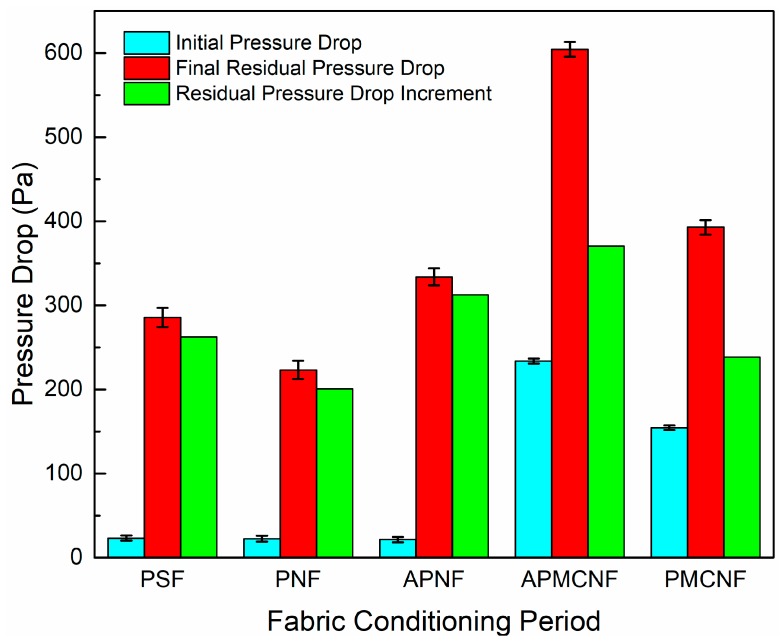
The final residual resistance and the initial residual resistance of the five filter media during the fabric conditioning period.

Figure 9 illustrates the initial filtration resistances, the final residual resistance drops, and the increments in the residual resistance drop of the five filter media in the aging phase. For the two surface filtration media, the initial filtration resistances and the final residual resistance drops were greater than those in the remaining three depth filtration media. Out of the five filter media, APMCNF filter media showed following characteristics: the highest initial filtration resistance, final residual resistance drop, and residual resistance drop increment. For PMCNF filter media, the increment in the residual resistance drop was less than that of APNF and PSF filter media. These results indicate that smaller the average pore size of the filter media, faster would be the increasing rate of the residual resistance drop of the filter media. In particular, when dust entered and deposited inside the depth filter media, the fiber diameter of the depth filter media further affected the dynamic filtration resistance of the filter media by influencing the deposition rate of dust.

**Figure 10:**
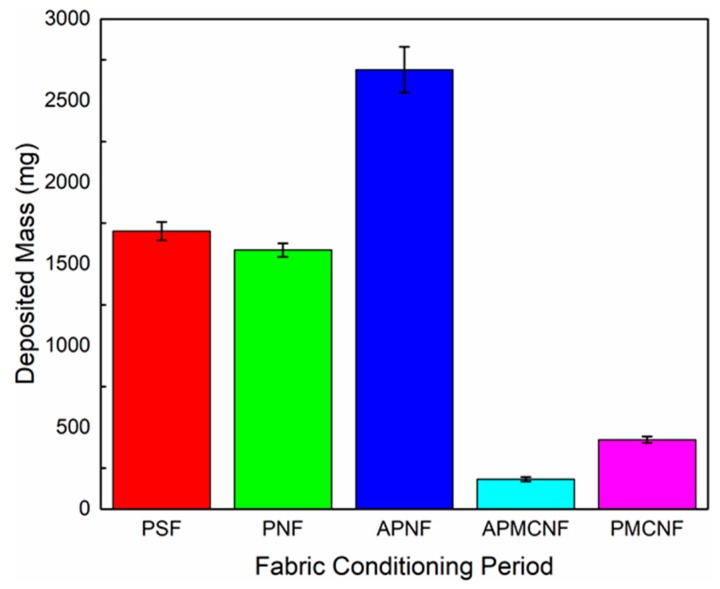
The mass gain of the five filter media during the fabric conditioning period.

Figure 10 illustrates the mass gain of filter media after the aging phase. The mass gains of various filter media were as follows: APNF > PSF > PNF > PMCNF > APMCNF. The mass gain of the depth filtration media was significantly greater than that of the surface filtration media. Therefore, the amount of dust deposited in the depth filtration media was much greater than that deposited on the surface of the membrane-coated filter media. For the depth filtration media, the experimental results indicate that the dynamic filtration resistance increases mainly because of the following reasons: (i) the deposition of dust inside the filter media and (ii) the formation of a dust cake. The experimental results further indicate that smaller the average pore size of the depth filtration media, greater would be the deposition rate of dust in the depth filtration media. A larger fiber diameter was more conducive for the deposition of dust and the formation of a dust cake inside the depth filtration media. For the surface filtration media, the experimental results indicate that the mass gain of APMCNF filter media was the least; however, the initial filtration resistance, the final residual resistance drop, and the increment in the residual resistance drop of APMCNF filter media were greater than those of PMCNF filter media. Based on the experimental results of the mass gain for the surface filtration media in the aging phase, it was further proved that the increase in the residual resistance drop of the surface filtration media was due to the blockage of dust in the pores of the filter membrane. Smaller the average pore size of the surface filtration media, easier would be the irreversible blockage of dust in the micropores of the surface filtration media and faster would be the increasing rate of dynamic filtration resistance of the surface filtration media. Moreover, smaller the average pore size of the surface filtration media, smoother would be the surface filtration media. Furthermore, the dust was stripped very thoroughly under the action of a cleaning airflow, and the mass gain of the surface filtration media was smaller in magnitude.

**Figure 11:**
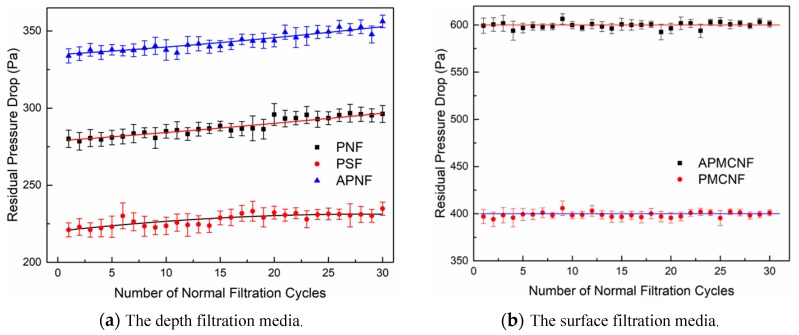
The changes in the residual resistance drop of the five filter media in the fabric recovery period.

Figure 11 illustrates the residual resistance drop that occurred in the five kinds of filter media during the recovery phase. Figure 11a illustrates that the residual resistance drop of the three kinds of depth filtration media increased with an increase in the filtration cycles that occurred during the recovery phase. In the 30 normal filtration cycles, the residual resistance drops of the three depth filtration media increased in the following order: APMCNF > PSF > PNF. In the recovery phase, the increasing rates of residual resistance drop of the three depth filtration media were obviously lower than those in the aging phase. This indicates that after providing aging treatment to the depth filtration media, the foundation of the dust cake improved gradually inside the depth filtration media. As a result, the formation rate of a dust cake became slower, and the deposition rate of dust decreased in the depth filtration media. Therefore, the residual resistance drops of the three depth filtration media increased slowly. During the recovery phase, the increments in the residual resistance drop of the three depth filtration media were as follows: 12.1 Pa (PSF), 10.3 Pa (PNF), and 17.3 Pa (APNF). The increments in the residual resistance drop and the increase rate of dynamic filtration resistance of the three depth filtration media were similar and much smaller than those in the aging phase. This indicates that for the depth filtration media, the average pore size and the fiber diameter greatly influenced the dust deposition rate at the initial stage of a dynamic filtration process. With the progress in dynamic filtration, the size and the distribution of filter pores in the depth filtration media changed with the deposition of dust inside the filter media. This weakened the influence of the average pore size and the fiber diameter of depth filtration media on the dust deposition rate.

As shown in Figure 11b, the residual resistance drops of the two surface filtration media were always observed at a relatively stable level in 30 normal filtration cycles, which was consistent with the experimental results in reference [27]. In contrast, the residual resistance drop of APMCNF and PMCNF filter media were about 600 Pa and 400 Pa, respectively. This indicates that a dynamic equilibrium was established between the deposition and the stripping of dust, which accumulates on the surface of the two membrane-coated filter media. After the aging treatment, fine particles were deposited on the micropores of the surface membrane; these particles were steadily removed by the cleaning airflow. Moreover, residual resistance drop of surface filtration media was also stabilized.

**Figure 12:**
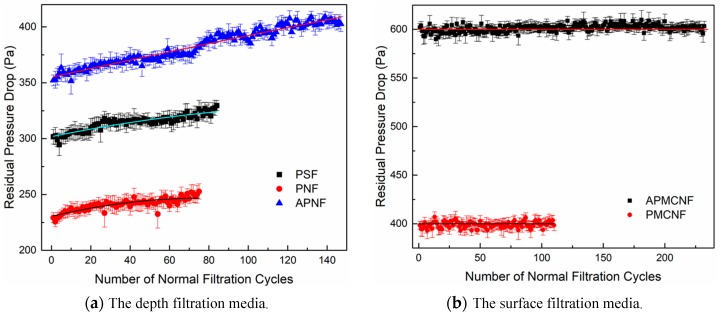
The changes in the average residual resistance drop of the five filter media during the performance test period.

Figure 12 shows the changes in the residual resistance drop of the five filter media during the measurement phase: the residual resistance drops of the three depth filtration media increased continuously when the filtration cycles were elongated after the aging and recovery phases. The residual resistance drops of the two surface filtration media remained relatively stable during the recovery and measurement phases, which occurred after the aging phase. The results of the entire dynamic filtration test of the five filter media showed that in the initial filtration stage (Figure 7), the increasing rate of the residual resistance drop was the fastest when a clean filter media was transformed into a dust-containing filter media (this conclusion can be obtained by comparing Figure 7, Figure 11 and Figure 12.). With the progress of the dynamic filtration process, there was a gradual improvement in the structure of a dust cake that was formed inside the depth filtration media. Moreover, a dynamic equilibrium was reached between the following two processes: (i) the blockage of membrane pores by dust and (ii) the removal of dust layer formed on the membrane of the surface filtration media. Consequently, the increasing rate of the residual resistance drop of the surface filtration media remained relatively stable. In the entire dynamic filtration test, the residual resistance drop of the depth filtration media increased steadily. At the end of the measurement phase, the final residual resistance drop of APNF filter media was greater than 400 Pa, indicating that it was greater than the final residual resistance drop of PMCNF filter media. It can be inferred that when the depth filtration media was used for long-term dynamic filtration, the residual resistance drop would eventually surpass that of the surface filtration media; moreover, the dynamic filtration resistance of the depth filtration media would increase at a rate faster than that of surface filtration media.

#### 3.2.2. Dynamic Filtration Efficiency

**Figure 13:**
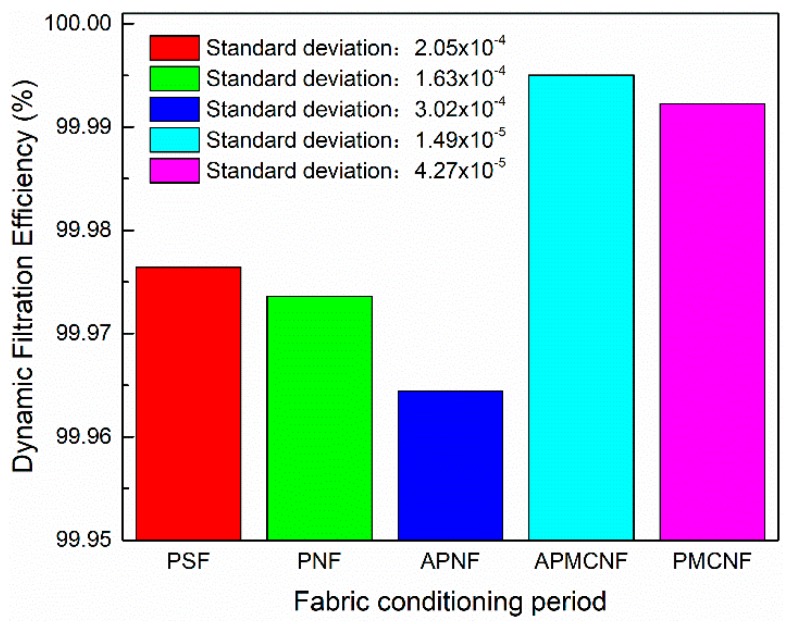
The dynamic filtration efficiencies of the five filter media during the fabric conditioning period.

**Figure 14:**
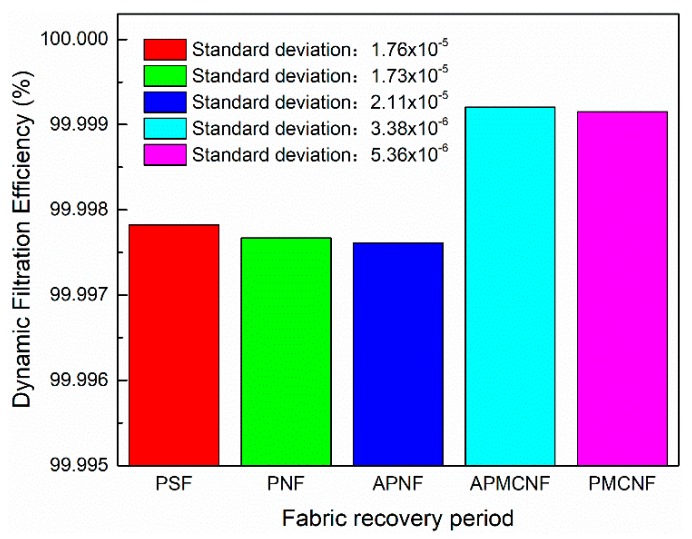
The dynamic filtration efficiencies of the five filter media during the fabric recovery period.

**Figure 15:**
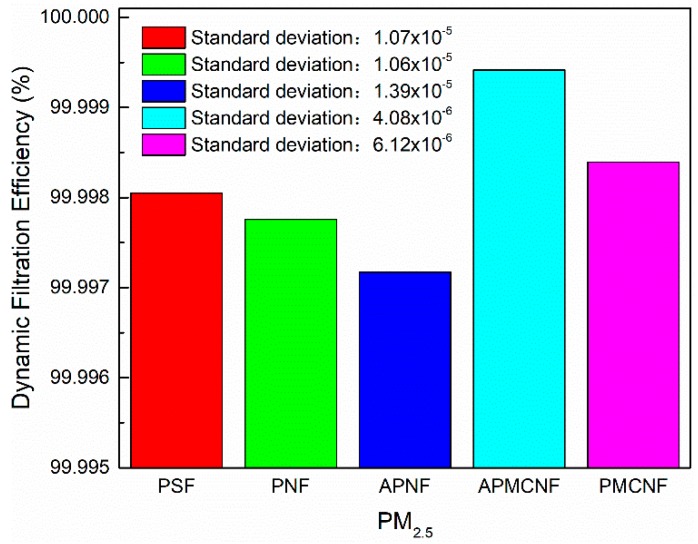
The dynamic filtration efficiencies of the five filter media for PM_2.5_ during the performance test period.

**Figure 16:**
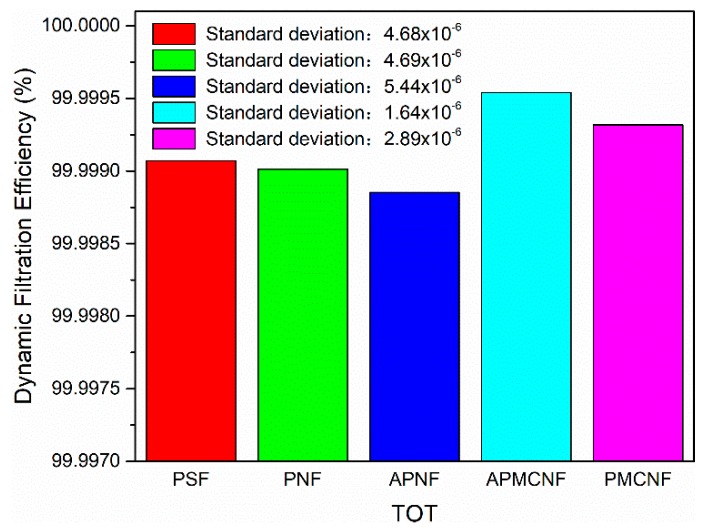
The dynamic filtration efficiencies of the five filter media for TOT during the performance test period.

As shown in Figure 13, Figure 14, Figure 15 and Figure 16, the dynamic filtration efficiencies of the five filter media were determined by performing dynamic filtration tests. Figure 13 and Figure 14 showed the dynamic filtration efficiencies of the five filter media in the aging and recovery phases, respectively. Figure 15 and Figure 16 illustrate the dynamic filtration efficiencies of PM_2.5_ and TOT for the five filter media during the measurement phase. The experimental results indicate that dynamic filtration efficiencies of the five filter media increased with the progression of the dynamic filtration process. In the entire dynamic filtration tests, the dynamic filtration efficiencies of the five filter media were greater than 99.96%. Compared to the static filtration efficiencies of the five filter media (Figure 6), the filtration efficiencies of the clean filter media were the lowest for a life cycle of filtration. Compared to the static filtration tests, the differences between the filtration efficiencies of the depth filtration media and the surface filtration media were greatly reduced during the dynamic filtration test. This indicates that dust deposition and dust cake formation were essential to improve the filtration efficiency of depth filtration media. At different phases of a dynamic filtration process, the dynamic filtration efficiencies of the five filter media were as follows: APMCNF > PMCNF > PSF > PNF > APNF. The order of dynamic filtration efficiencies of the five filter media was opposite to that of their average pore size, that is, smaller the average pore size of the filter media, greater was the dynamic filtration efficiency of the filter media.

In the depth filtration media, the “Dust filter Dust” filtration mechanism of was implemented. According to this mechanism, greater the deposition of dust in the filter media, better would be the structure of a dust cake and greater would be the dynamic filtration efficiency. In contrast, the dynamic filtration resistance of a filter media increases with the deposition of dust inthe filter media. Based on the above inferences, we propose the following conclusion: under the same experimental conditions, faster the increasing rate of dynamic filtration resistance of the depth filtration media, faster would be the deposition rate of dust inthe filter media, and higher would be the dynamic filtration efficiency of the filter media. In the dynamic filtration experiments, we found that the increasing rate of the residual resistance drop was the fastest for the APNF filter media, followed by the PSF filter media, and the PNF filter media. Therefore, the deposition rate of dust was the highest for the APNF filter media, followed by the PSF filter media and the PNF filter media. This inference has been confirmed in the mass gain of the filter media, which occurred during the aging phase. The magnitude of dynamic filtration efficiencies of the three filter media were in the following order: APNF > PSF > PNF; this finding was not compliant with the actual situation of the test (PSF > PNF > APNF). The reason for the above discrepancy was the fact that in the dynamic filtration process, the structure of the dust cake formed inside the depth filtration media was destroyed by the compressed air, enabling dust to penetrate through the depth filtration media. Some research studies [28] have shown that in a filtration cycle, dust leakage occurs mainly in a short period of time after cleaning the filter media; moreover, dust penetration rate decreases rapidly as the dust layer is repaired on the surface of the filter media. After cleaning the depth filtration media, the surface and the fabric structure of the depth filtration media were exposed to an airflow of dust. Larger the average pore size of the depth filtration media, easier was it for the dust to penetrate into the depth filtration media; moreover, larger the fiber diameter, lower was the filtration efficiency of the fabric structure of the depth filtration media. Therefore, the APNF filtration media has the highest increase rate of residual resistance drop; however, its dynamic filtration efficiency was the lowest.

In the surface filtration media, the microporous membrane was used as a barrier for filtering the particles (sieving effect). The size and the distribution of membrane pores in the filter media were used to determine the performance of filtration. For the surface filtration media, the experimental results of the dynamic filtration efficiency were compliant with the laws used to perform experiments of dynamic resistance. These experimental results were different from those obtained for the depth filtration media. Smaller the average pore size of the surface filtration media, higher would be the dynamic filtration resistance and the dynamic filtration efficiency.

In the aging phase, dynamic filtration efficiencies of the three depth filtration media were in the range of 99.96–99.98%. For the two surface filtration media, the dynamic filtration efficiencies were greater than 99.99% in the aging phase. During the recovery phase, the dynamic filtration efficiencies of all the five filter media were greater than 99.99%. In the measurement phase, the dynamic filtration efficiencies of the five filter media were greater than 99.997% for PM_2.5_, and the dynamic filtration efficiencies of the five filter media were greater than 99.998% for TOT. In the entire dynamic filtration tests, the dynamic filtration efficiencies of the two surface filtration media were always greater than those of the three depth filtration media. This implies that dynamic filtration efficiencies of the surface filtration media were more stable in magnitude. In the entire dynamic filtration process, the standard deviations of the dynamic filtration efficiencies of the three depth filtration media were always greater than those of the two surface filtration media. For the dynamic filtration efficiencies of dust, the standard deviations of the five filter media were as follows: APNF > PNF> PSF > PMCNF > APMCNF, which were in the same order as the average pore size of five filter media. It can be concluded that smaller the average pore size of the filter media, smaller would be the standard deviation in the dynamic filtration efficiency; therefore, the dynamic filtration efficiency of the filter media would be more stable.

## 4. Conclusions

In this study, the static filtration performance of clean filter media was investigated, which was used to study the effects of filter pore size and fiber diameter on the filtration performance of the fabric structure of the filter media, so as to provide basic data and theoretical support for the study of the influence of filter pore size and fiber diameter on the dust deposition characteristics and dynamic filtration performance of the dust-containing filter media (the fabric structure + the dust cake). It was found that the average pore size was the main factor affecting the filtration performance of the fabric structure of the surface filtration media: the smaller the average pore size of the filter media, the higher would be the filtration efficiency and greater would be the filtration resistance of the fabric structure. For the depth filtration media, smaller the average pore size and the fiber diameter of the filter media, greater would be the filtration resistance and higher would be the filtration efficiency and precision of the fabric structure.

By performing the dynamic filtration tests, we concluded that the average pore size and the fiber diameter of the depth filtration media affected the dynamic filtration resistances and the dynamic filtration efficiencies by influencing the deposition rates of dust in the depth filtration media. Smaller the average pore size of the depth filtration media, easier was it for the dust to be deposited inside the depth filtration media and more compact would be the formation of a dust cake inside the depth filtration media; moreover, greater would be the amount of dust deposition inside the depth filtration media, and faster would be the increasing rate of dynamic filtration resistance. With a steady increase in the fiber diameter, the probability of dust deposition would increase tremendously on the surface of the fiber and between the fibers. These conditions were conducive for the deposition of dust inside the depth filtration media. Filter pore size and fiber diameter can significantly affect the dust deposition rate in the initial stage of dynamic filtration of the depth filtration media, and then the effect is gradually weakened with the progress of the dynamic filtration process. In a dynamic filtration process, the structure of a dust cake inside the depth filtration media and the accumulation of a dust layer on the surface of the depth filtration media were destroyed by compressed air. At this time, the influence of filter pore size and fiber diameter on the filtration efficiency of fabric structure determined the dynamic filtration efficiency of depth filtration media within a short period of time after cleaning. Therefore, depth filtration media with larger fiber diameter had lower filtration efficiency and filtration resistance of its own fabric structure, but the increasing rate of dynamic filtration resistance was higher, and the dynamic filtration efficiency was also lower.

The dynamic filtration resistances and the dynamic filtration efficiencies of the surface filtration media were related to the average pore size of the filter media. Smaller the average pore size of the surface filtration media, easier would be the irreversible blockage of dust in the membrane micropores of the surface filtration media and faster would be the increasing rate of dynamic filtration resistance; moreover, higher the dynamic filtration efficiency, more stable would be the dynamic filtration process. In addition, smaller the average pore size of the surface filtration media, larger would be the dynamic equilibrium value of the residual resistance drop of the filter media, and shorter would the filtration cycle of the surface filtration media.

## Figures and Tables

**Table 1:** The performance parameters of the five tested filter media.

Filter Media	Fiber Diameter (μm)	Grammage (g·m^−2^)	Thickness (mm)	Fiber-Filling Rate (%)	Average Pore Size (μm)
PSF	16.0 ± 0.5	500.0 ± 0.6	1.80 ± 0.02	19.40 ± 0.25	18.26 ± 0.63
PNF	16.0 ± 0.3	500.0 ± 0.7	1.80 ± 0.05	19.40 ± 0.23	22.49 ± 0.76
APNF	16.0 ± 0.4(95 wt%) + 28.0 ± 0.6(5 wt%)	500.0 ± 0.8	1.80 ± 0.04	19.40 ± 0.21	22.52 ± 0.81
APMCNF	16.0 ± 0.4(95 wt%) + 28.0 ± 0.6(5 wt%)	500.0 ± 0.8	1.80 ± 0.03	/	0.42 ± 0.08
PMCNF	16.0 ± 0.3	500.0 ± 0.6	1.80 ± 0.04	/	1.05 ± 0.09
